# Divergences of the *RLR* Gene Families across Lophotrochozoans: Domain Grafting, Exon–Intron Structure, Expression, and Positive Selection

**DOI:** 10.3390/ijms23073415

**Published:** 2022-03-22

**Authors:** Shanshan Yao, Jiulin Chan, Yue Xu, Shimei Wu, Linlin Zhang

**Affiliations:** 1 College of Life Sciences, Qingdao University, Qingdao 266071, China; 17805421231@163.com (S.Y.); shimeiwu2016@126.com (S.W.); 2 CAS and Shandong Province Key Laboratory of Experimental Marine Biology & Center of Deep Sea Research, Center for Ocean Mega-Science, Institute of Oceanology, Chinese Academy of Sciences, Qingdao 266071, China; chanjiulin@qdio.ac.cn (J.C.); xuyue@qdio.ac.cn (Y.X.); 3 Laboratory for Marine Biology and Biotechnology, Qingdao National Laboratory for Marine Science and Technology, Qingdao 266071, China; 4 College of Marine Science, University of Chinese Academy of Sciences, Beijing 100049, China

**Keywords:** *RIG-I*-like receptor, lophotrochozoan, innate immune, molecular evolution, gene expression, domain grafting, exon–intron structure, positive selection

## Abstract

Invertebrates do not possess adaptive immunity but have evolved a variety of unique repertoires of innate immune sensors. In this study, we explored the immune diversity and specificity of invertebrates based on the lophotrochozoan *RLRs*, a major component in antiviral immune recognition. By annotating *RLRs* in the genomes of 58 representative species across metazoan evolution, we explored the gene expansion of *RLRs* in Lophotrochozoa. Of note, the N-terminal domains of lophotrochozoan *RLRs* showed the most striking diversity which evolved independently by domain grafting. Exon–intron structures were revealed to be prevalent in the domain grafting of lophotrochozoan *RLRs* based on an analysis of sibling paralogs and orthologs. In more than half of the cases, the mechanism of ‘exonization/pseudoexonization’ led to the generation of non-canonical N-terminal domains. Transcriptomic studies revealed that many non-canonical *RLRs* display immune-related expression patterns. Two of these *RLRs* showed obvious evidence of positive selection, which may be the result of host defense selection pressure. Overall, our study suggests that the complex and unique domain arrangement of lophotrochozoan *RLRs* might result from domain grafting, exon–intron divergence, expression diversification, and positive selection, which may have led to functionally distinct lophotrochozoan *RLRs*.

## 1. Introduction

The immune system has long been known for its remarkably rapid evolution due to strong selective drivers, such as fast-evolving pathogens [1,2]. Lophotrochozoa is one of the most species-rich superphyla, including mollusks, segmented worms, and other invertebrates. Despite lacking adaptive immunity, these animals exhibit a high level of biodiversity and a wide range of ecological adaptations, including adaptation to a complex pathogenic environment [3]. Innate immunity is not only the main defense mechanism of crown rotors against pathogens, but also the most conservative defense mechanism of multicellular organisms against pathogens [4,5]. Innate immunity is characterized by a rapid, nonspecific response to infection and injury. Invertebrates lacking immunoglobulin-mediated adaptive immunity have evolved a variety of broad, unique, and complex repertoires of innate immune sensors [6,7]. An alternative strategy for invertebrates is the large-scale expansion and diversification of multigene families encoding genome-encoded pattern recognition receptors (PRRs) [8,9,10].

Animals distinguish self from non-self by using a variety of PRRs [11,12]. PRRs interact with pathogen-associated molecular patterns (PAMPs) and damage-associated molecular patterns (DAMPs) [13,14,15]. PRR-binding events typically trigger signaling cascades that lead to the transcription of immune effector genes encoding products such as antibacterial and antiviral proteins, leading to the production of proinflammatory cytokines and the death of host cells [16,17]. PRRs are divided into the following major categories: cytoplasmic NOD-like receptors (*NLRs*), membrane-bound Toll-like receptors (*TLRs*), C-type lectin receptors (*CLRs*), scavenger receptors, AIM2-like receptors (*ALRs*), and RIG-I-like receptors (*RLRs*) [14].

*RLRs* are a family of three DExD/H box-containing RNA helicases [18]. In vertebrates, *RLRs* include retinoic acid-inducible gene I (*RIG-I*), melanoma differentiation-associated gene 5 (*MDA5*), and laboratory of genetics and physiology 2 (*LGP2*) [19]. *MDA5* and *RIG-I* contain two N-terminal caspase (cysteine-dependent aspartate-specific proteases) recruitment domains (CARDs), followed by an intermediate DEAD/DEAH box helicase domain (DEXDc + HELICc domains) and a C-terminal RIG-I_C-RD domain [20]. Helicases use the energy provided by the hydrolysis of ATP to catalyze the unwinding of nucleic acid duplexes. CARD is a structural domain composed of amphipathic α-helices and is predicted to function through protein–protein interactions with apoptotic and antiapoptotic proteins. In contrast, *LGP2* lacks CARDs and retains the DEAD/DEAH box helicase domain and RIG-I_C-RD domain. The simultaneous presence of CARD and RNA helicase domains in a single molecule, as observed in *MDA5* and *RIG-I*, is unique [21]. According to previous studies, this unique mechanism for preventing viral infection is caused by the shuffling of domains [21]. Recent studies have shown that the domain organization of *RLRs* in early animals differs from that in vertebrates. Four types of structural compositions of RLR proteins are found in the brachiopod *Lingula anatina* [22]. The N-terminus of these *LanRLRs* presents different domains, including a death effector domain (DED) and caspase catalytic (CASc) domains, in addition to CARD. Only one CARD has been found at the N-terminus of the *RLRs* in cnidarians [23]. However, the evolutionary lineage of *RLRs* across all metazoans is unknown, especially the evolutionary pattern of their domain architecture.

As one of the main cytoplasmic PRRs, *RLRs* are responsible for the intracellular dsRNA sensor induced by type I IFN [20,24]. An analysis of *MDA5* or *RIG-I* knockout mice revealed that this TLR-independent pathway is central to innate immunity against viral infection [25,26,27]. Moreover, as both *MDA5* and *RIG-I* are IFN-stimulated genes, a positive feedback loop that generates a potent antiviral state is established [28,29]. The prototypical *RLRs* contain three types of functional domains: the two CARD domains at the N-terminus that are responsible for downstream signaling transduction, the DEXDc domain and the HELICc domain in the center that are responsible for RNA recognition, and the C-terminal RIG-I_C-RD domain that assists in pathogen recognition by binding to specific viral RNAs [30]. *MDA-5* and *RIG-I* interact with the CARD domain of the mitochondrial protein IFN-β promoter stimulator-1 (*IPS-1*, also known as *MAVS*, *VISA*, and *CARDIF*). Thereafter, TNF-receptor-associated factor-3 (*TRAF-3*) is recruited, and *TRAF* family member-associated NF-κB-activator binding kinase-1 (*TBK1*) and inducible IκB kinase (*IKK*) are activated [31,32,33]. These kinases phosphorylate *IRF-3* and *IRF-7* and activate *NF-κB*, which translocates to the nucleus to induce type I IFN expression [34]. *MAVS*-dependent *RLR* signaling has been shown to be involved in viral immunity in mollusks [35]. Further, as *LGP2* lacks CARDs, it does not trigger immune responses; however, it can regulate *RIG-I* and *MDA5* signaling [36,37]. The regulatory function of *LGP2* is attributed to its retained helicase domain and RD [38,39]. 

Evolutionary studies have painted a complex picture of how *RLRs* emerge, and their functional diversity. Previous studies suggested that full-length *RLRs* are a vertebrate-specific evolutionary novelty, although the building blocks of *RLRs* may have been present in closely related prevertebrate animals [21,40]. These studies differ in the ordering of gene duplication events leading to the three *RLRs* present in mammals; however, the studies ultimately concluded that *RLR* evolution was driven by a complex series of domain grafting and gene fusion events in CARD domains. Based on recent studies, the *RLR*-based immune system is not vertebrate-specific but originates in the earliest multicellular animals [41]. *RLRs* functionally diversified through a series of gene duplication events followed by protein coding changes, which modulate the RNA-binding properties of different *RLRs* by altering key contact residues within the C-terminal RD [41]. There is strong evidence that *RLRs* are involved in a long-term evolutionary arms race with viral RNA molecules, suggesting that the structure of viral RNA may have shaped the evolution of animal innate immunity [41]. Although there is no consensus on the exact evolutionary history of *RLRs*, these receptors (and/or their building blocks) have been reported to originate early in metazoan evolution [41,42] and, most importantly, are evolutionary hot spots [42]; this is illustrated by the lineage-specific loss of *RLR* genes in many species. For example, most mammals possess *RIG-I*; however, *RIG-I* is lost in at least one mammalian species, the Chinese tree shrew [43]. Interestingly, with the loss of *RIG-I*, both *MDA5* and *LGP2* have undergone strong positive selection in Chinese tree shrews, and positively selected sites in *MDA5* endowed the substitute function for the lost *RIG-I* [44]. Adaptive evolution analysis of the *RLR* gene family in reptiles, birds, and other chordates also revealed that purification selection was dominant [45,46]. However, an overview of large-scale metazoan macroevolution is lacking.

Here, we explored the immune diversity and specificity of the *RLR* gene family in lophotrochozoans. Briefly, we annotated 227 *RLR* genes from 58 species across the metazoan phylogeny, with emphasis on the molecular evolutionary dynamics of *RLRs* in lophotrochozoans. Further, we explored their phylogeny and domain composition, which revealed that lophotrochozoan *RLRs* exhibited highly diverse and complex N-terminal domain integration. We also established an exon–intron structure to investigate the molecular mechanism underlying domain grafting. Evolutionary selection signals and tissue and infective gene expression levels were also calculated. Overall, a comprehensive molecular evolution analysis of the *RLR* gene family, which is not only associated with rapid domain grafting but also with the potential for an immune response by positive selection, was carried out.

## 2. Results

### 2.1. Identification of the RIG-I-like Receptor Repertoires

**Figure 1:**
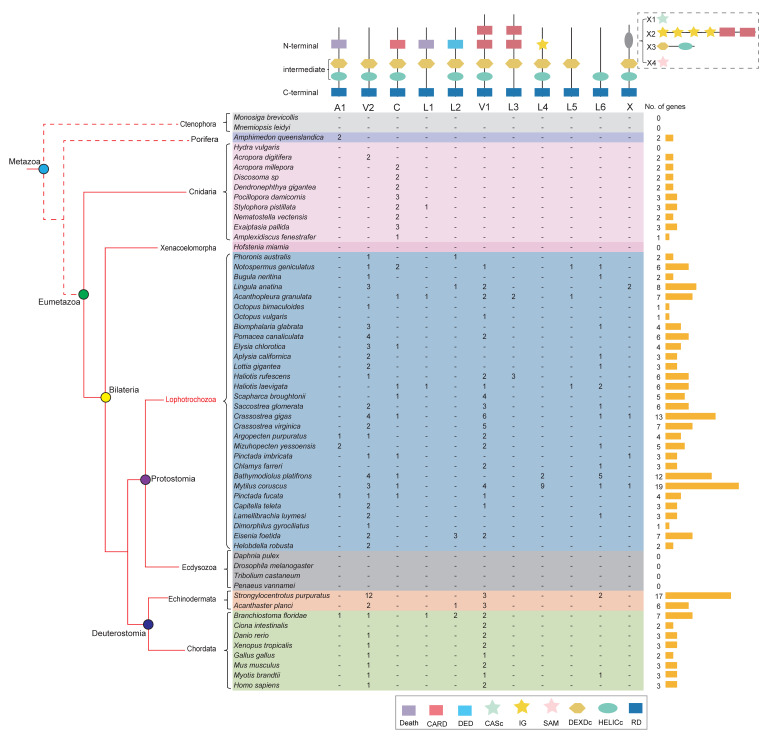
Comparison of gene families encoding *RLR* immune receptors in representative animals across metazoans. Domain architecture in the lophotrochozoans is more complex than that in other clades. Species colors represent different phyla. Short lines indicate none of the *RLRs* in the subtype were annotated in this species. Phylogenetic relations among species are indicated by the red cladogram on the left of the table, and dashed lines represent unresolved phylogenetic positions for ctenophores and sponges. The column on the right counts the total number of *RLR* genes in each species and draws a yellow column chart. *RLR* diagrams show death family domain in purple, CARD domain in red, DED domain in light blue, CASc domain in light green, IG domain in light yellow, SAM domain in pink, DEXDc domain in yellow, HELICc domain in green, and RD domain in blue. Specifically, the top diagrams show A1, Amphimedon-like type 1; V1, vertebrate-like type 1; V2, vertebrate-like type 2; C, cnidaria-like type; L1, lophotrochozoa-like type 1; L2, lophotrochozoan-like type 2; L3, lophotrochozoan-like type 3; L4, lophotrochozoan-like type 4; L5, lophotrochozoan-like type 5; and L6, lophotrochozoan-like type 6. X implies four specific structural features (X1–X4).

To investigate the composition difference in the *RLR* gene family across all metazoans, we first annotated 227 *RLRs* in the genomes of 58 representative species with different evolutionary positions (Supplementary Tables S1 and S2). Two *RLR* genes were identified in the sponge of the phylum Porifera (*Amphimedon queenslandica*), suggesting potential origins of *RLRs* in early metazoans (Figure 1). The *RLR* gene was completely lost in arthropods of the 58 species investigated (Figure 1), which is consistent with the results of previous studies [41]. Two or three *RLRs* in most (88%) of the chordates were annotated, while seven *RLRs* were annotated in *Branchiostoma floridae*. Compared with chordate animals, *RLRs* were extensively expanded in most (60%) lophotrochozoans. Further, 13 *RLRs* in the genome of *Crassostrea gigas*, 12 in *Bathymodiolus platifrons*, and 19 in *Mytilus coruscus* were predicted. The expansion of *RLRs* was also identified in the echinoderm *Strongylocentrotus purpuratus*.

**Figure 2:**
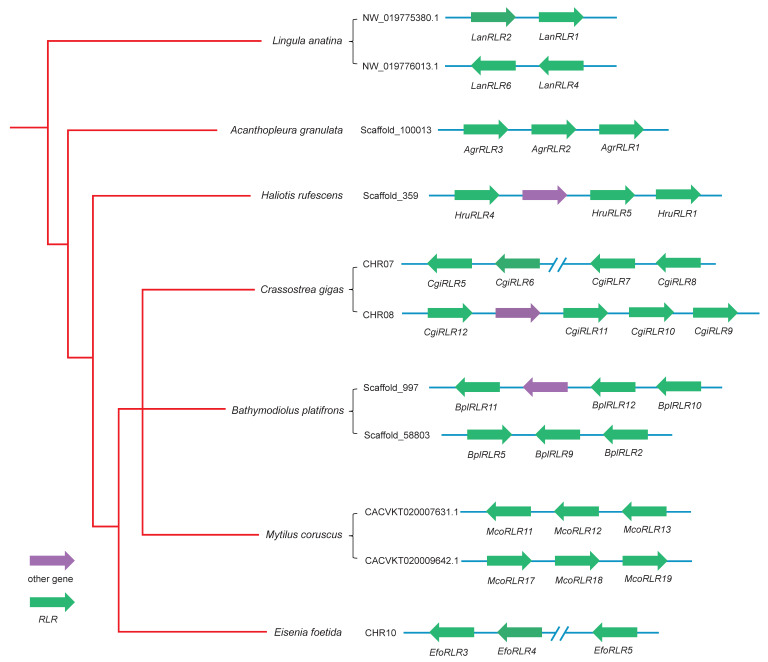
Local tandem duplication of *RLRs* in the seven representative lophotrochozoan species. Green arrowheads indicate *RLR* genes and their transcriptional direction; purple arrowheads indicate other genes. Phylogenetic relations among species are indicated by the red cladogram on the left.

Further genomic distribution exploration found that the expansions of the lophotrochozoan *RLRs* can be attributed to multiple local tandem duplication events (Figure 2 and Supplementary Table S3). The tandem duplication phenomenon is the most noticeable in the bivalve species with extensive expansion of *RLRs*. Specifically, 8 of 13, 6 of 12, and 6 of 19 *RLRs* were found to be linked in tandem arrays in the bivalves *C. gigas*, *B. platifrons*, and *M. coruscus*.

### 2.2. RLR Domain Annotation

Canonical *RLRs* have a typical C-terminal RNA recognition domain (RD) that binds viral RNA and N-terminal CARDs to interact with the signal adaptor. We proceeded to explore the domain architecture of the *RLRs*. With the inclusion of the canonical vertebrate types (V-type), we classified metazoan *RLRs* into 11 major types based on their domain architecture (Figure 1). Most (83%) vertebrates expressed three *RLRs*, including two V1 types (*RIG-I* and *MDA5*) and one V2 type (*LGP2*). In the ancestral branch, Porifera (*A. queenslandica*), two A1-type *RLRs* with death as the N-terminal domain were annotated. Nine of the ten cnidarians were identified to possess two or three *RLRs*, and most (89%) cnidarian anthozoan *RLRs* were C (cnidarian-type), with only one CARD domain in the N-terminus. The types of *RLRs* that only occur in one species are classified as group X, which includes four species (X1–X4). Intriguingly, the domain architecture of *RLRs* showed the most striking diversity in lophotrochozoans, which contain the eleven divided RLR types. The N-termini of those eleven *RLR* types include: canonical V1 and V2 types, the A1 type of the death domain, the C type of the N-terminal CARD, the six L types (L1–L6) obtained from lophotrochozoans, and the X type. This observation may reflect a high level of domain grafting, resulting in significant expansion of *RLRs* in lophotrochozoans.

### 2.3. Phylogenetic Distribution of Three Discrete Domains

**Figure 3:**
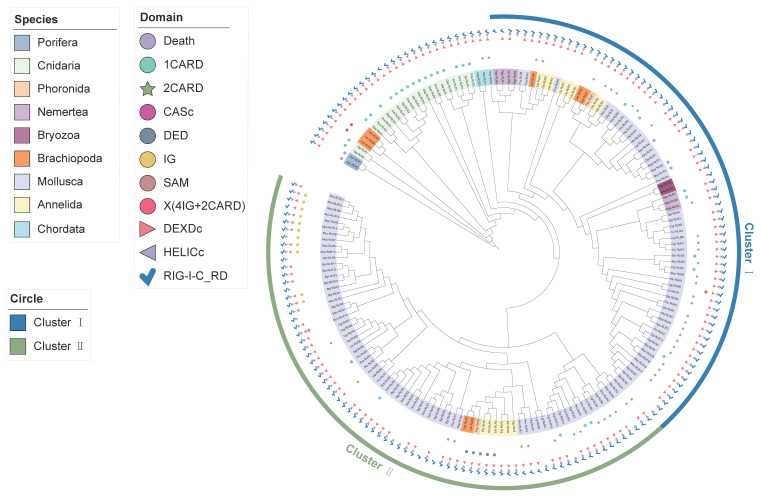
Phylogenetic analysis of *RLRs* based on C-terminal conserved domain RIG-I_C-RD. Maximum likelihood trees were constructed using IQ-TREE. The species used for the tree generation were 30 lophotrochozoans (as shown in Figure 1), the poriferan *A. queenslandica*, cnidaria, and the chordate *Homo sapiens*. The lophotrochozoan *RLRs* can be majorly classified into two divergent classes, Cluster I and Cluster II. The background color on the gene name represents the phylum to which it belongs, as shown in the legend ‘Species’. The different colored shapes in the outer circles represent different types of domains, as shown in the legend ‘Domain’.

To determine the molecular evolutionary history of the *RLR* gene family in these species, we traced the phylogenetic origins of three representative domains, including RIG-I_C-RD, the DEAD/DEAH box helicase domain, and the N-terminal domain. First, a phylogenetic tree was constructed using the C-terminal conserved domain RIG-I_C-RD coding sequences of all metazoan *RLRs* (Figure 3). This tree suggested that ancestral *RLRs* duplicated in lophotrochozoans, with no *RIG-I*/*MDA5*/*LGP2* divergence detected in this phylum. Further, most lophotrochozoan *RLRs* were found to be derived from the ancestral *RLR* gene and were divided into two groups (Cluster I and Cluster II) (Figure 3). Cluster I was clustered with the ancestral porifera and vertebrate *RLRs*, indicating that lophotrochozoan *RLRs* in Cluster I might have originated from the same ancestor as those in vertebrates. Three types of N-terminal domains were detected in the *RLRs* of Cluster I, including the canonical CARD and ancestral death type. Compared with lophotrochozoan *RLRs* in Cluster I, diversity and plasticity in the N-terminal domain architecture were observed in *RLRs* in Cluster II. Six of the seven N-terminal domains (Death, CARD, CARD-CARD, (immunoglobulin) IG, DED, (Sterile alpha motif) SAM) in this cluster were annotated. In Cluster II, the *RLRs* with N-terminal IG and DED domains were found to be independently clustered, which suggested that the L2 (DED) and L4 (IG) types of *RLRs* were independent in lophotrochozoans. 

**Figure 4:**
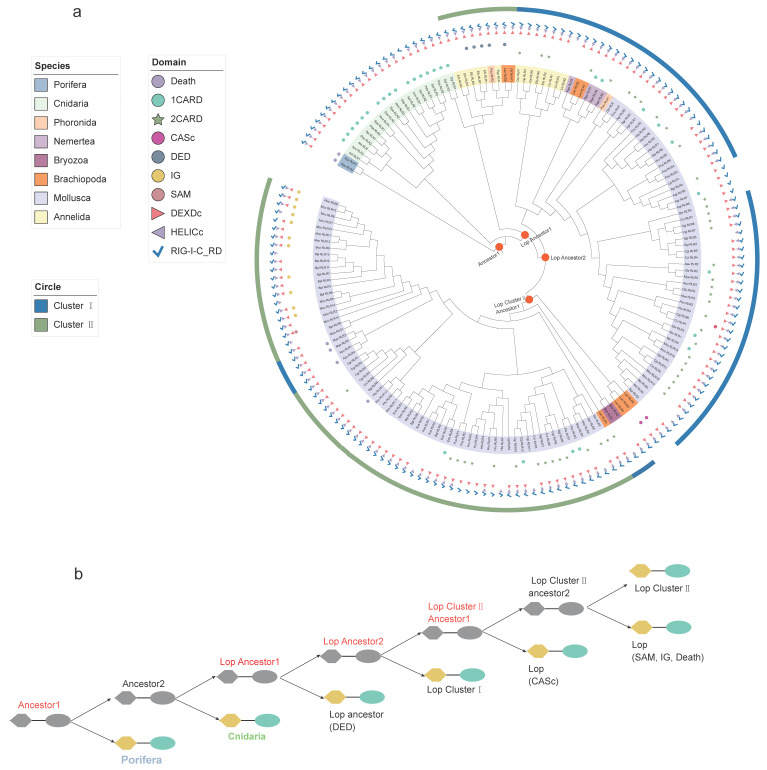
The evolution of intermediate helicase domains (DEXDc + HELICc domains) of the lophotrochozoan *RLRs*. (**a**) Phylogenetic tree constructed with the maximum likelihood method showing the evolution of DEXDc + HELICc domains of *RLRs* in loptrochozoans. The different background colors of the gene names represent the phyla to which they belong, as shown in the legend ‘Species’. The different colored shapes represent different domains, as shown in the legend ‘Domain’. The blue arc in the outermost circle represents Cluster I, and the green arc represents Cluster II. The orange circles on the branches marked on the tree correspond to the evolutionary divergent nodes as shown in (**b**). (**b**) A schematic diagram of the evolution of the intermediate helicase domains of lophotrochozoan *RLRs* based on the phylogenetic tree. Lop stands for the lophotrochozoan. Solid black lines indicate duplication events.

We proceeded to investigate the evolutionary history of the *RLR* gene family based on the intermediate helicase domain (DEXDc + HELICc domains) (Figure 4). Generally, the phylogenetic tree topology of the helicase domain is very similar to that of the RIG-I_C-RD phylogenetic tree, indicating that the integration of RIG-I_C-RD and helicase domains originated before the divergence of metazoans. In contrast to the phylogenetic tree based on RIG-I_C-RD, the lophotrochozoan *RLRs* with an N-terminal DED domain belonged to Cluster II in the helicase tree instead of Cluster I. In addition, the RLRs with the N-terminal death domain were within Cluster I in the helicase tree but belonged to Cluster II in the RIG-I_C-RD tree. These results support the hypothesis that linked domains of intermediate helicases and RIG-I_C-RD have ancient origins in metazoans; however, fusion events of the two domains in *RLRs* with an N-terminal DED or death domain might independently occur later under certain selection pressures. 

**Figure 5:**
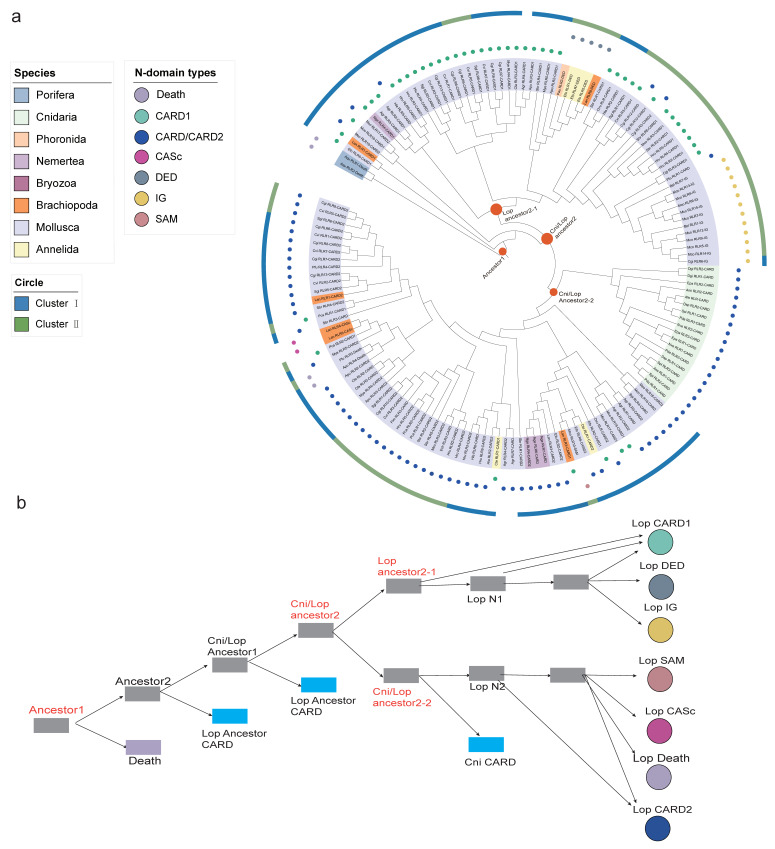
The evolution of N-terminal domains (CARD, DED, IG, death, CASc, SAM) of lophotrochozoan *RLRs*. (**a**) Phylogenetic tree constructed with the maximum likelihood method showing the evolution of N-terminal domains of *RLRs* in lophotrochozoans. The different background colors of the gene names represent the phyla to which they belong, as shown in the legend ‘Species’. The different colored circles represent different domains, as shown in the legend ‘Domain’. The blue arc in the outermost circle represents Cluster I, and the green arc represents Cluster II. The orange circles on the branches marked on the tree correspond to the important nodes in (**b**). (**b**) A schematic diagram of the evolution of the N-terminal domains of *RLRs* in lophotrochozoans based on the phylogenetic tree. Cni stands for Cnidaria, and Lop stands for the lophotrochozoan. Ancestor1 indicates an ancestor where a duplication produced the first N-terminal death domain and a common ancestor of all other N-terminal domains. Cni/Lop ancestor2 indicates an ancestor where a duplication occurred that produced different N-terminal domains. Lop N1 represents the first domain of the N-terminal RLR, and Lop N2 represents the second. Solid black lines indicate duplication events. Colors of N-terminal domains correspond to color labels in the trees in (**a**).

To clarify the evolutionary relationship of the diverse N-terminal domains of *RLRs* in lophotrochozoans, we conducted phylogenetic analysis using sequences of N-terminal domains of *RLRs* in lophotrochozoans, the ancient death domain in porifera, and the CARD domain in cnidaria (Figure 5). The two different CARD domains (CARD1 and CARD2) in the typical type of lophotrochozoan *RLRs* were separately extracted and used for phylogenetic analysis. Based on the phylogenetic tree (Figure 5a), the evolutionary histories of N-terminal domains displayed overall different patterns compared to the helicase and RIG-I_C-RD trees, especially for *RLRs* with lophotrochozoan-specific N-terminal domains. Such pheromones suggest the occurrence of multiple independent fusion events in lophotrochozoan *RLRs*. The simultaneous presence of death and helicase domains occurred in porifera, which might be the ancient state in metazoans. Further, lophotrochozoan *RLRs* with the death domain may not be orthologs of the poriferan *RLRs*; instead, these *RLRs* shared the same ancestor with cnidarian *RLRs* containing the CARD domain. Based on the evolutionary history of the CARD domain, cnidarian C-type *RLRs* encode only one CARD instead of two CARD domains in canonical V1-type *RLRs*. The second CARD domain of lophotrochozoan *RLRs* clustered with the ancestral cnidarian CARD, indicating that the fusion of the CARD1 and CARD2 domains in the common ancestor of the lophotrochozoan ancestor might have occurred through an independent process. Of the N-terminal domains of L (lophotrochozoan)-type *RLRs*, DED and IG clustered with the CARD1 branch, while SAM and CASc clustered with the CARD2 branch. These results indicate that the highly diverse and dynamic N-terminal domains in lophotrochozoan *RLRs* could have independently emerged from domain grafting. 

### 2.4. Intron–Exon Structure Analysis of Lophotrochozoan RLRs with Diverse N-Terminal Domains

**Figure 6:**
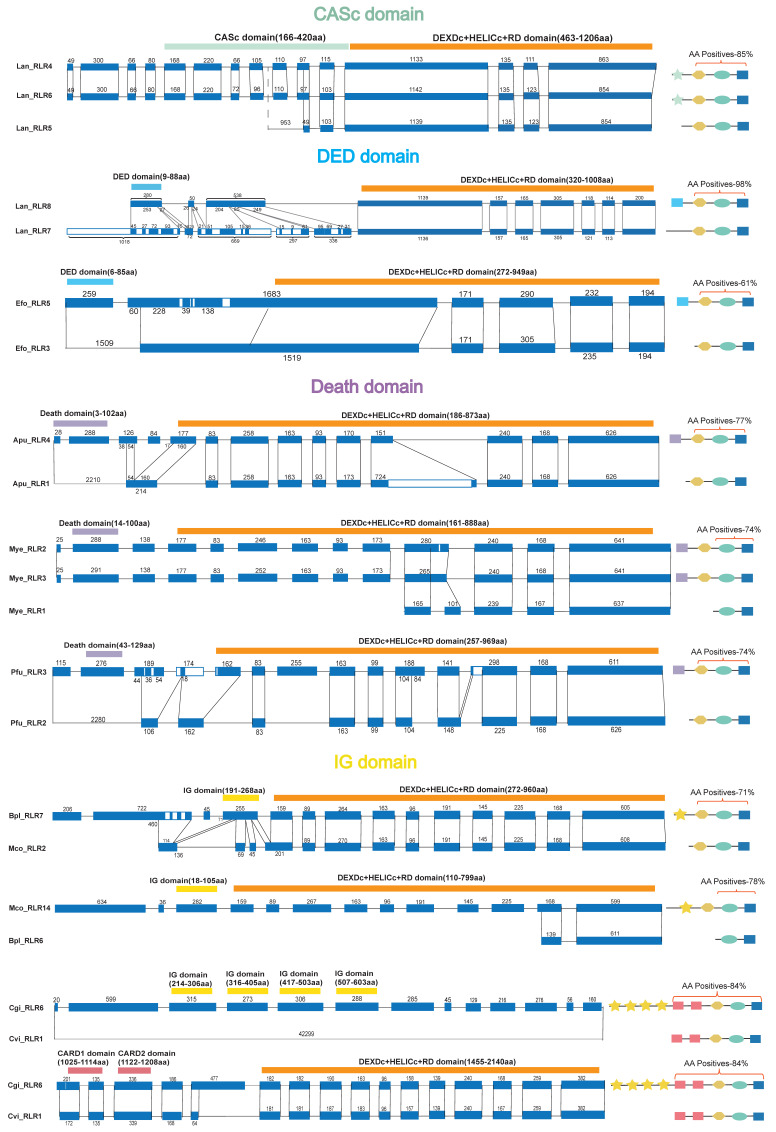
Exon–intron structure analysis of *RLRs* with diverse N-terminal domains in lophotrochozoans. The exon–intron structures (**left**) and the schematic diagrams of the domain composition (**right**) of the *RLRs* with non-canonical N-terminal domains of nine groups of homologous genes. The blue rectangle represents the exon, and the solid line represents the uncoded sequence in the alignment. The number marks the length of the exon or unencoded sequence. White bars in exons depict the indels that have resulted from insertion/deletion events.

Previously, exon shuffling was believed to be one of the major forces driving domain grafting [47]. Accordingly, we aimed to determine whether exon–intron structure-related mechanisms contribute to the highly diverse N-terminal domains of lophotrochozoan-specific *RLRs*. Our hypothesis was tested by identifying sibling paralogs/orthologs with the highest sequence similarity based on the RIG-I_C-RD phylogenetic tree and comparing their exon–intron architecture (Figure 6). We first manually optimized all the shown gene models by transcriptomic read mapping to make sure the sequences of these sibling paralogs/orthologs had high confidence. Exon–intron structure divergence was found to be prevalent in the *RLRs* in all N-specific domains studied, including domains CASc, DED, Death, and IG. Further, the exon–intron structure was classified into three types, including ‘gain/loss of exon/intron,’ ‘exonization/pseudoexonization,’ and ‘intraexonic insertion/deletion’ [48]. Among the examples of the nine homologous *RLR* groups with a difference in the N-terminal domain in Figure 6, ‘gain/loss of exon/intron’ was found to be prevalent in four cases, ‘exonization/pseudoexonization’ in five cases, and ‘intraexonic insertion/deletion’ in only one case.

The type ‘gain/loss of exon/intron’ is a process through which an entire partial exon/intron is inserted/deleted. For the sibling paralogs *LanRLR7* and *LanRLR8* in *L. anatina*, the domain structure of *LanRLR7* is DEXDc-HELICc-RD and does not encode the interaction N-terminal domains. Comparatively, an exon gain event with 253 amino acids was inferred in *LanRLR8*, leading to an additional N-terminal DED domain. This gain of exon did not cause a shift in the reading frame. The other corresponding exonic sequences of this sibling paralog could still be aligned with high confidence, except for the gained exon, with 98% sequence similarity at the amino acid level. The structure type of ‘gain/loss of exon/intron’ was found in the domain grafting of death in the *Mizuhopecten yessoensis* sibling paralogs *MyeRLR2, MyeRLR3*, and *MyeRLR1*. The same structure type was found in the domain grafting of the IG domain in the lophotrochozoan-specific sibling ortholog pairs *BplRLR7*-*McoRLR2* and *McoRLR14*-*BplRLR6*. The mechanism of ‘exonization/pseudoexonization’ is a process that leads to interchanges between exonic and non-exonic sequences. For the sibling ortholog pairs *CgiRLR6* and *CviRLR1* in oyster *RLRs*, exonization of 13 exons was characterized in *CgiRLR6*, which led to a lophotrochozoan-specific *RLR* with four IG domains in the N-terminal sequence. A similar phenomenon was found in the sibling paralogs *LanRLR4*-*LanRLR5*-*LanRL6* and *EfoRLR5*-*EfoRLR3*, and the sibling ortholog *PfuRLR3*-*ApuRLR4*, which led to the domain grafting of CASc, DED, and death in the lophotrochozoan-specific *RLRs*, respectively (Figure 6). Finally, intraexonic insertion/deletion was found in the domain shuffling of DED in the sibling paralog *LanRLR7*-*LanRLR8*. Of note, the three types of structural divergence were not mutually exclusive. 

By analyzing six cases of sibling paralogs and three cases of sibling orthologs, we found that the most predominant type of mechanism for structural variation in the *RLR* genes with lophotrochozoan-specific N-terminal domains was ‘exonization and pseudoexonization,’ which was observed in five of the nine cases studied. The second most predominant was ‘gain/loss of exon/intron,’ which was observed in four cases. Notably, the three types of exon–intron structure-related mechanisms were prevalent in the canonical V1- and C-type *RLRs*. In contrast to lophotrochozoan *RLRs* encoding diverse N-terminal domains, the type ‘loss/gain of exon/intron’ is most predominant in these canonical *RLRs* instead of the type ‘exonization/intronization’ (Supplementary Figure S1). 

### 2.5. Expression Profiles of the Lophotrochozoan RLRs 

**Figure 7:**
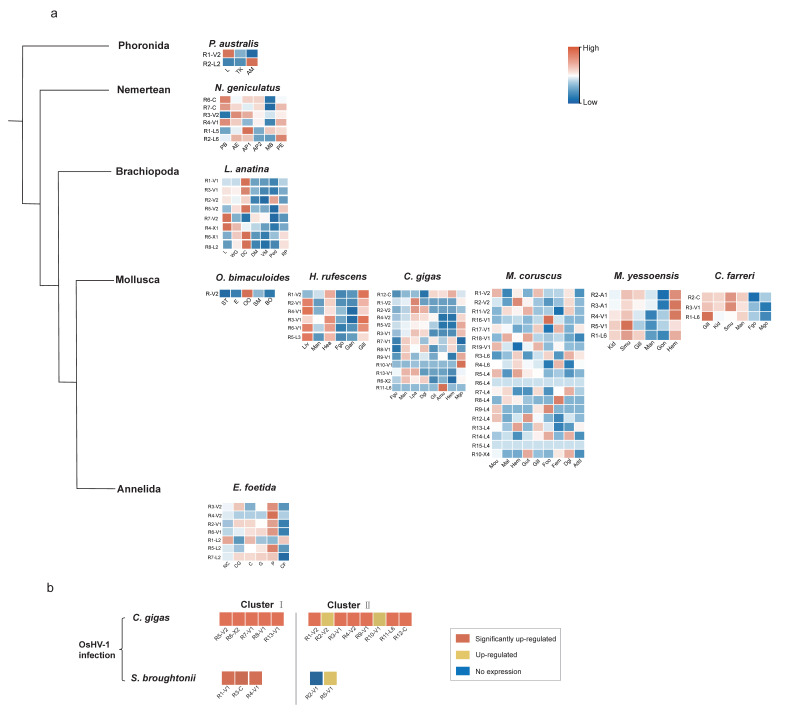
Expression patterns of *RLR* genes among different tissues and under virus infection. (**a**) Transcriptome expression heatmaps of *RLRs* of different tissues in ten Lophotrochozoa species. Ampulla (AM), trunk (TK), proboscis (PB), anterior end (AE), anterior part1 (AP1), anterior part2 (AP2), mid-body (MB), posterior end (PE), lophophore (L), whole gut tissue (WG), digestive cecum (DC), dorsal mantle (DM), ventral mantle (VM), pedicle (Ped), regenerated pedicle (RP), statocyst tissue (ST), eye (E), olfactory organ (OO), skin from mantle (SM), brain tissue from optic lobe (BO), liver (Liv), mantle (Man), heart (Hea), female gonad (Fgo), adult female ganglion (Gan), gill (Gill), labial palp (Lpa), digestive gland (Dgl), adductor muscle (Amu), hemocyte (Hem), male gonad (Mgo), mouth (Mou), male gonad (Mal), foot (Foo), female gonad (Fem), adductor muscle (Add), kidney (Kid), striated muscle (Smu), gonad (Gon), nerve cord (NC), chlorogog gut (CG), crop (C), gizzard (G), pharynx (P), coelomic fluid (CF). Heatmap displays the expression level of *RLR* genes. (**b**) Transcriptional change of the *RLRs* under Ostreid herpesvirus-1 (OsHV-1) infection. The orange rectangle represents significantly upregulated *RLR* genes (Log2(FC) > 1.5); the yellow rectangle represents the upregulated *RLR* genes but not significantly; the blue rectangle represents the *RLR* genes that are not expressed.

As large expansions and highly diverse domain structures were observed in lophotrochozoan *RLRs*, we determined whether these lophotrochozoan *RLRs* are functional. We collected all published lophotrochozoan tissue transcriptome data from the NCBI GEO database (up to June 2021) and calculated the tissue expression levels of *RLR* genes in ten evolutionary representative species (Figure 7a and Supplementary Table S4), including the phoronidan *Phoronis austrailis*; the nemertean *Notospermus geniculatus*; the brachiopod *L. anatina*; the mollusks *Octopus bimaculoides*, *Haliotis rufescens*, *C. gigas*, *M. coruscus*, *M. yessoensis*, and *Chlamys farreri*; and the annelida *Eisenia foetida*. Among the 71 *RLRs* studied in the 10 species, 65 were found to be expressed in at least one tissue. Fourteen of the twenty *RLRs* encoding specific N-terminal domains showed expression based on the tissue expression profiles. This result suggests that most lophotrochozoan *RLRs*, both the canonical ones and those with various N-terminal domains, were potentially functional. 

Owing to the prevalence of expression in duplicated lophotrochozoan *RLRs* with diverse N-terminal domains, whether these genes retained the function of immune reorganization was unknown. To clarify whether such retention existed, we compared the expression levels of *RLRs* in immune-related and unrelated tissues (Figure 7a and Supplementary Table S4) and found both tissue-prevalent and specific patterns in lophotrochozoan *RLRs* with diverse N-terminal domains. In the bivalve *C. gigas*, most *CgiRLRs* with diverse N-terminal domains were found to be highly expressed in the labial palp, mantle, male gonad, and digestive gland. In the brachiopod *L. anatina*, *LanRLR8* with an N-terminal DED domain and *LanRLR6* with an N-terminal CASc domain were found to be significantly expressed in the digestive tissue. Molluscan *McoRLR10* with an N-terminal SAM domain and *McoRLR12* with an N-terminal IG domain were also highly expressed in the gut, and *McoRLR10* was highly expressed in the digestive gland (Figure 7a and Supplementary Table S4). These results indicate that *RLRs* might be important in mucosal immunity. However, molluscan *MyeRLR2* and *MyeRLR3* with an N-terminal death domain showed the highest expression levels in hemocytes, suggesting their potential function in hemocyte-mediated immunity. Many duplicated lophotrochozoan *RLRs* with special N-terminal domains were upregulated in both the digestive gland and hemocytes, such as *McoRLR5* and *McoRLR13* with an N-terminal IG domain, which were highly expressed in hemocytes (Figure 7a and Supplementary Table S4). Taken together, these results suggest that many *RLRs* composed of diverse N-terminal domains are highly expressed in immune-related tissues and might play a significant role in innate immune recognition.

We determined whether the duplicated lophotrochozoan *RLRs* with diverse N-terminal domains would be upregulated under viral challenges. We searched for virus or virus-related PAMP infection transcriptomes in the NCBI GEO database (up to June 2021), found two published databases from bivalves *C. gigas* and *Scapharca broughtonii*, and calculated their expression profiles (Figure 7b). A total of 11 of the 13 *RLRs* in *C. gigas* were significantly upregulated (Log2 (FC) > 1.5) during oyster herpes virus infection. Of these *RLRs*, *CgiRLR6* encodes a lophotrochozoan-specific N-terminal IG domain. Similarly, *S. broughtonii SbrRLR3* encoding only one CARD at the N-terminus was significantly upregulated (Log2 (FC) > 1.5) under virus challenge. These results suggest that lophotrochozoan *RLRs* with special domains may play an important role in antiviral immune recognition. 

### 2.6. Evidence of Positive Selection in Lophotrochozoan RLRs with Diverse N-Terminal Domains 

As innate immune receptors are responsible for severe diseases, *RLRs* must rapidly evolve and are thus subjected to positive selection pressures [44]. We determined whether the molecular evolution of the duplicated lophotrochozoan *RLRs* with diverse N-terminal domains was driven by natural selection. Accordingly, a positive analysis was performed on lophotrochozoan *RLRs* with diverse N-terminal domains and gene expression levels enriched in the immune-related tissues mentioned above. We used the RIG-I_C-RD domain of *RLRs* for selection analysis, as this part is the virus recognition domain. 

**Figure 8:**
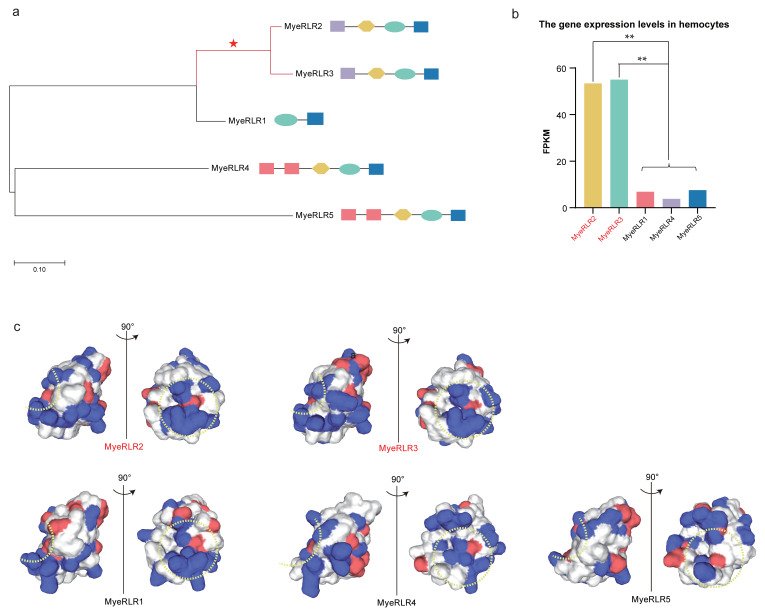
Positive selection analysis of representative *RLR* genes. (**a**) Evolutionary relationships of *MyeRLRs*. The branches with *dN*/*dS* (ω) values > 1.0 are marked with red lines. (**b**) The expression level of five *MyeRLRs* in hemocytes. One-way ANOVA showed that the expression levels of *MyeRLR2* and *MyeRLR3* were significantly (*p*-value < 0.01, marked with **) more upregulated than those of other *MyeRLRs*. (**c**) Electrostatics of the RNA-binding surfaces of the *MyeRLR* RIG-I_C-RDs. The determination of RNA-binding sites refers to previous studies [49]. Positively charged surfaces are colored blue, and negatively charged surfaces are red. Dotted yellow line indicates RNA-binding loop.

We performed positive selection analysis on five *RLRs* of *M. yessoensis*, all of which were in Cluster I of the phylogenetic tree (Figure 3). Positive selection signals (ω = 521.37) could be identified in branches, including *MyeRLR2* and *MyeRLR3* (Figure 8a). Both genes encoded the N-terminal death domain. We further reconstructed the 3D structures of the five *MyeRLRs* (Figure 8c). Based on the results, the surface of the RNA-binding region of *MyeRLR2* and *MyeRLR3* is positively charged, which is consistent with human *RIG-I* [41]. Although *MyeRLR1* is positively charged, its N-terminus has no domain, which may prevent its function. However, the surface of the RNA-binding region of *MyeRLR4* and *MyeRLR5* has a less positive charge. 

Of note, the selection analysis was consistent with the gene expression patterns in *RLRs* of *M. yessoensis*. Both *MyeRLR2* and *MyeRLR3* showed a significantly higher expression in the immune-related tissue hemolymph (Figure 8b). The lophotrochozoan *RLRs* with diverse N-terminal domains might interact with other adaptors without a CARD domain, triggering the unique lophotrochozoan cascade in antiviral immunity. The selection results suggest that lophotrochozoan-specific *RLRs* were under rapid positive selection, indicating that these unique *RLRs* might play important and novel roles in innate immunity.

## 3. Discussion

Interaction between pathogens and hosts leads to a dynamic evolutionary arms race. Invertebrates, which lack adaptive immunity, evolved a variety of broad, unique, and complex repertoires of innate immune sensors. In this study, we explored the diversity and specificity of invertebrate innate immune recognition in the lophotrochozoan *RLR* gene family and identified the diversity of *RLRs* in lophotrochozoans, which is mainly reflected in the divergence of the N-terminal domains. By exploring the molecular evolutionary mechanism driving the diversity of domain arrangement in lophotrochozoan *RLRs*, we found that it might be due to rapid domain grafting, exon–intron structural divergence, expression diversification, and positive selection. To the best of our knowledge, this is the first systematic study of the molecular evolution of *RLRs* in lophotrochozoans. 

One of the most interesting findings of this study is that lophotrochozoan *RLRs* represent the successful use of genetic linkages of N-terminal domains to expand and diversify the immune repertoire. The invertebrate immune system is innate and encoded in the germline. Extensive expansion of immune receptors has been proposed to reveal an alternative mechanism for the diversity and specificity of innate immune recognition in the absence of an adaptive immune system [8,9,10]. In our study, the *RLR* gene families experienced expansion, aligning with previous immune receptor studies in amphioxi [9]. Importantly, the domain arrangement was found to be highly diverse in the duplicated lophotrochozoan *RLRs*. Manual correction of all gene models with non-canonical N-terminal domains was performed, and their domain arrangements were examined, which indicated that our data can be used for further phylogenetic and molecular evolution studies. Therefore, gene models were predicted with high confidence, arguing against gene modeling errors as an explanation for our results.

In vertebrates, the N-terminal CARD domain of *RIG-I* and *MDA5* interacts with the CARD domain of the mitochondrial protein *MAVS* for signal transmission [31,32,33,50]. In invertebrates, the protein interaction between the N-terminus of canonical *RLR* receptors and the adaptor *MAVS* is conserved in the mollusk *C. gigas* [35]. Of note, non-canonical N-terminal domains, including the death, DED, CASc, IG, and SAM domains, were also observed in lophotrochozoan *RLRs*. Among them, the death and DED subfamily, together with CARD, constitutes the death domain (DD) superfamily. By mediating homotypic interactions within each domain subfamily, these proteins play important roles in the assembly and activation of apoptotic and inflammatory complexes [51,52]. The CASc domain represents the C-terminal conserved domain found in caspases, mainly from animals. Caspases are mainly involved in mediating cell apoptosis and are recruited as apoptosis initiators that trigger the apoptosis process, and as effectors of apoptosis [53,54]. The CASc domain is also reported to be involved in inflammatory processes [55]. Previous studies suggested that another important intracellular immune receptor, *NLR* (nucleotide oligomerization domain (NOD)-like receptor), recruited the apoptosis-related domains pyrin and baculovirus inhibitor repeats during evolution, leading to the control of the activation of inflammatory caspases in animals [56,57]. We hypothesized that lophotrochozoan *RLRs* with a CASc domain might be involved in apoptosis, inflammation, or pyroptosis. Molecules with IG-like domains are involved in a variety of immunological functions, including adaptive immune receptors, innate immune molecules, and accessory molecules [58]. Previous studies suggested that diverse forms of IG-containing molecules and their specificity of immune function in non-self recognition or interaction with endogenous molecules are remarkable [59]. One of the well-known examples is the IG domain’s variation in the snail fibrinogen-related proteins that exhibits different forms of somatic variation [60]. Although the C-terminal domain of *RLR* is responsible for initial pathogen recognition, the N-terminal domains are required for downstream signaling [24,30]. Novel immune response diversity and specificities might thus be acquired by integrating non-canonical domains into *RLRs* in lophotrochozoans. These new integrated non-canonical protein domains might play an unidentified role in innate immunity or host defense. 

The phylogenetic results suggest that the lophotrochozoan *RLRs* integrated with non-canonical N-terminal domains were distributed unevenly across the *RLR* phylogeny with the dominant clade in Cluster II, and most were species-specific. Most lophotrochozoan *RLRs* clustered with vertebrate *RIG-I* instead of *MDA5*/*LDP2* and were divided into two clusters. The domain composition showed that the functions of the *RLRs* in Cluster II were more diverse than those in Cluster I. Previous studies have shown that in addition to *RLRs* [21], the other two gene families encoding immune receptors, *NLR* and *TLR*, underwent massive species-specific expansions and domain shuffling in various lineages [61]. In the NACHT protein family, a diversity of N-terminal domains, including death, CARD, DED, BIR, and PYRIN domains, was found. Among them, three types of domain combinations have emerged multiple times in different lineages, including death-NACHT-LRR, CARD-NACHT-LRR, and PYRIN-NACHT-LRR [61]. Studies on the TIR protein family have shown that across the metazoans, the N-terminal domain connected to TIR has the IG, death, and SAM domains, and IG-TIR has emerged multiple times in different lineages [61]. In contrast, in the specific domains of the N-terminus of *RLRs* (except CARD), the death, DED, and IG domains have emerged multiple times in different lineages, while the CASc and SAM domains have only been found in one species. Two separate domains of *TLR*, the TIR domain and the LRR domain, have been found to have a domain combination phenomenon. The combination of the P-TIR and P-LRR domains occurred in non-bilateria, while the early combination of the V-TIR and V-LRR domains for V-TLR occurred after the divergence of bilateria and non-bilateria [62]. Generally, it is speculated that the combination of the above domains is not random but follows certain rules, which might be selective pressure exerted by pathogens in a specific environment [61,62]. In addition, the phenomenon of domain shuffling in killer cell Ig-like receptors (*KIRs*) is expressed on the surface of NK cells, which leads to the production of new *KIRs* [63].

Interestingly, the exon–intron structure seems to significantly contribute to the integration of non-canonical N-terminal domains in the lophotrochozoan *RLRs*. The structure of the RLR protein is relatively simple in ancestral metazoan porous animals and cnidarians. However, in lophotrochozoans, the domain arrangement of the RLR protein showed a complex and diverse pattern with the addition of the second CARD (CARD1) and non-canonical N-terminal domains. Deletion and incorporation of specific domains have become prevalent. Based on our results, the diversity of the N-terminal domains in lophotrochozoan *RLRs* should be attributed to domain shuffling after gene duplication. As the domains are often correlated with exon boundaries, exon shuffling is believed to be one of the major forces driving domain shuffling [47]. Our study indicates that intronization/exonization is the main driving force of exon shuffling. A similar phenomenon of exon shuffling was observed in the *KIR* gene family. For *KIR* genes, the exon–intron structure correlates with the four main parts: the first three parts comprise an Ig domain, D0, D1, and D2, and the last part comprises the stem (S), transmembrane (TM), and cytoplasmic (CYT) domains [63,64]. A previous study speculated that, in addition to domain shuffling’s *KIR* being more favored by natural selection, another important reason for domain shuffling is that certain introns are hot spots for recombination [63]. For the *RLR* gene, almost all specific domains were found to be fully encoded by only one exon (Figure 6), which also creates an environment in which exon shuffling occurs.

Finally, as positive selection signals and immune-related expression patterns were detected independently in different clades of lophotrochozoan non-canonical *RLRs*, these domain fusions may have a selective advantage for the organisms [62,65]. The possibility of domain recombination to achieve functional diversity has been mentioned in many evaluations [63,66,67], and the diversity of the N-terminal domain may affect the specificity of ligand binding [66]. The study of the function of abaecin through exon shuffling suggests the role of exon shuffling in buffering the loss-of-function mutations in a gene [67]. However, at present, sufficient immune-related transcriptome data are unavailable to verify whether the new lophotrochozoan *RLRs* have new functions. Further, whether proteins with the same domain architecture share similar functions in different species is still unknown. Some examples including those from the families discussed here suggest that this notion does not always hold true. For instance, although Drosophila Toll-like receptors mainly perform roles in embryonic development, their mammalian homologs are key regulators of immune responses [68,69]. Therefore, extrapolation of protein function based on the domain architecture must be performed very carefully. 

## 4. Materials and Methods

### 4.1. Data Collection 

To identify the *RLR* genes, the genome and protein sequence datasets of 58 metazoan species at different evolutionary nodes were collected from the National Center for Biotechnology Information (NCBI) (https://www.ncbi.nlm.nih.gov/, accessed on 1 June 2021) or from other databases (Supplementary Figure S2; Table S1). Transcriptome data for *RLR* gene expression analysis were obtained from the NCBI Sequence Read Archive (SRA) database (https://www.ncbi.nlm.nih.gov/sra/, accessed on 1 June 2021). Multiple biotic stress data included PRJNA450478 (*S. broughtonii* after Ostreid herpesvirus-1 infection), and PRJNA146329 (*C. gigas* larvae infected with OsHV-1). Adult tissue data included PRJNA393252 (*P. australis*); PRJNA393252 (*N. geniculatus*); PRJNA286275 (*L. anatina*); PRJNA658966 (*O. bimaculoides*); PRJNA488641 (*H. rufescens*); PRJNA146329 (*C. gigas*); PRJNA578350 (*M. coruscus*); PRJNA185465 (*C. farreri*); PRJNA259405 (*M. yessoensis*); PRJNA608692 (*E. foetida*). 

### 4.2. RLR Gene Identification and Phylogenetic Analysis

We used domain prediction and a sequence homology search of the *RLR* genes in 58 metazoan species (Figure S2). First, a local version of HMMER version3.1b2 (Howard Hughes Medical Institute; Cambridge, UK, 2015) [70], available from http://hmmer.org/download.html (accessed on 1 June 2021), was used to identify the *RLR* genes by screening the RIG-I_C-RD (PF11648) domain on the genomes of all species. Second, regions of each genome potentially harboring *RLR* genes were identified using TBLASTN (National Center for Biotechnology Information, Bethesda, MD, USA) [71] with RLR proteins from model organisms as query sequences. When we used the above two methods to find the loss of RLRs in some species, we used PSI-BLAST (http://blast.ncbi.nlm.nih.gov/, accessed on 4 March 2022) to further confirm this conclusion. Third, according to the structural characteristics of *RLRs* in a broad sense, we used the visualization software SMART (http://smart.embl-heidelberg.de/, accessed on 1 September 2021) [72] to screen the *RLR* genes of each species containing an intermediate DEAD/DEAH box helicase domain and a RIG-I_C-RD domain. Finally, we corrected some *RLR* genes with suspected domain deletions or special domains using local GeneWise software (https://www.ebi.ac.uk/Tools/psa/genewise/, accessed on 1 September 2021) [73] and transcriptome read mapping, which greatly ensures the accuracy of each N-terminal-specific *RLR* gene model studied.

The highly conserved sequences of the RIG-I_C-RD domain of *RLRs* identified by PFAM HMM were used to conduct the phylogenetic analyses in the present study. The protein sequences encoding the RIG-I_C-RD domain were aligned using the L-INS-I strategy in the local MAFFT v7.310 (Kazutaka Katoh, Osaka, Japan, 2013) [74]. Maximum likelihood trees were generated using IQ-TREE v1.6.12 (Bui Quang Minh, ANU(Australian National University), Australia, 2011) [75], which could automatically test and select the best alternative model. In order to test branch reliability, we adopted the fast bootstrap method with 1000 replicates, which can also be integrated into IQ-TREE. Trees were handled using iTOL v6 (https://itol.embl.de/, accessed on 1 November 2021). In addition, in order to explain the overall evolution of *RLRs*, the N-terminal domain coding sequences and intermediate helicase domain (DEXDc + HELICc domains) coding sequences were extracted to construct phylogenetic trees according to the same method.

### 4.3. Identification of RLR Homologs and Analysis of Gene Structures

First, the local BLAST version2.9.0 (National Center for Biotechnology Information, Bethesda, MD, USA) was used for homologous gene pair identification by reciprocal BLAST searching on the retrieved datasets (Figure S2). Then, the above results were confirmed by phylogenetic analysis of the *RLR* gene family. However, the homologous gene pairs found by this method were often too limited, so we only used the method of phylogenetic analysis to identify *RLR* gene homologous pairs containing special lophotrochozoan *RLRs*.

To determine whether paralogous or orthologous genes have diverged in exon–intron structure, we compared their genomic sequences. Two paralogs or orthologs were regarded as structurally divergent if they had different numbers of exons, or if they had the same number of exons but the lengths of at least one pair of homologous exons were different. To understand the underlying mechanisms of structural divergence, we generated pairwise alignments for each gene pair, using the corresponding mRNAs as guidance. Intraexonic insertion/deletion was deduced when an indel was found within the aligned homologous exons. Exon/intron gain/loss was inferred if an orphan exon/intron was the result of exon duplication, exon shuffling, exon scrambling, intron insertion, or intron deletion. Exonization/pseudoexonization was identified when the corresponding exonic and non-exonic sequences could be aligned with confidence [48].

### 4.4. Transcriptomic Analysis of Gene Expression

The FastxToolkit pipeline (http://hannonlab.cshl.edu/fastx_toolkit/index.html, accessed on 1 November 2021) was used to process the raw reads to evaluate sequencing quality and remove low-quality reads (length threshold < 50 bp and quality threshold < 20), adaptor sequences, poly-N, and known non-coding RNAs (Figure S2). Genome-based indexing and sam file generation were achieved using local Hisat2 version 2.1.0 (University of Texas Southwestern Medical Center, Dallas, TX, USA, 2017) [76] and Bowtie2 version 2.3.5.1 (University of Maryland, College Park, MD, USA, 2019) [77], and sam files were sorted using Samtools version 1.11 (Wellcome Genome Campus, Hinxton, Cambridgeshire CB10 1SA, UK) [78]. The obtained clean reads were then individually mapped to the genome of the respective species. Gene expression levels were measured by fragments per kilobase million (FPKM). The expression levels were quantified using Cufflinks version 2.2.1 (Harvard University, Cambridge, MA, USA) [79]. The differentially expressed *RLR* genes (DEGs) were identified with the edgeR tool of the R programming language with the threshold value |log2FC| ≥ 1.5 (multiple of fold change, FC: difference) and FDR ≤ 0.05. 

### 4.5. Positive Selection Analysis

Multiple sequence alignments of *RLRs* were performed using ClustalW (https://www.genome.jp/tools-bin/clustalw, accessed on 1 December 2021) with default parameters, and the resulting alignments were refined with trimAl version1.2(Centre for Genomic Regulation, Barcelona, Spain) [80] (Figure S2). Phylogenetic trees were constructed with ML analytical approaches based on MEGA7. The robustness of the inferred trees was assessed using bootstrapping with 1000 replicates in the phylogenetic tree. Phylogenetic trees were visualized using ITOL and used for subsequent positive selection analysis. 

PAML version4.9j (University of California, Berkeley, USA) [81] was used for comparing the rate per site of dN (nonsynonymous) to the rate per site of dS (synonymous) mutations. The recommended subset of four M-series models of M1a (nearly neutral), M2a (positive selection), M7 (beta), and M8 (beta and ω) coupled with Bayesian empirical Bayes (BEB) methods was implemented. The log-likelihood values (lnL) of M2aM1a and M8-M7 were from explicit tests for the presence of positively selected sites. The *p* values were corrected by a multiple testing correction method. Furthermore, the probabilities of sites under positive selection were assessed by their posterior probabilities calculated with the BEB method. The amino acid site was considered as a positively selected site if the value of *dN*/*dS* > 1 appeared in the LRT and the posterior probability exceeded 90%. Finally, SWISS-MODEL (http://swissmodel.expa-sy.org/, accessed on 10 December 2021) was used to locate and visualize the 3D structure of *RLRs*.

## 5. Conclusions

In this study, we systematically described the evolutionary history of the *RLR* gene family in lophotrochozoans by investigating their domain architecture, phylogeny, exon–intron structure, expression profiles, and selection patterns. Our study revealed many previously unknown N-terminal domain fusions in lophotrochozoan *RLRs*, which might result in the diversity and specificity of the innate immune response. We traced the exon–intron structure of these non-canonical lophotrochozoan *RLRs* and found that the mechanism of exonization/pseudoexonization might drive the formation of these *RLRs*. Many non-canonical lophotrochozoan *RLRs* exhibit positive selection signals and immune-related expression patterns, indicating that non-canonical lophotrochozoan *RLRs* might have a selective advantage for organisms in the innate immune response. Overall, our findings suggest that the complex and unique domain arrangement of lophotrochozoan *RLRs* might result from rapid domain grafting, exon–intron structural divergence, expression diversification, and positive selection, which may have led to functionally distinct paralogs or orthologs in the innate immune response. Our research provides new insights into the molecular evolution of innate receptors in invertebrates in the absence of antibody-mediated adaptive immunity.

## Data Availability

Not applicable.

## Supplementary Materials

The following supporting information can be downloaded at: https://www.mdpi.com/article/10.3390/ijms23073415/s1.
