# Supplementary material for: Divergences of the RLR Gene Families across Lophotrochozoans: Domain Grafting, Exon–Intron Structure, Expression, and Positive Selection
Source: Int J Mol Sci. 2022 Mar 22;23(7):3415. doi: 10.3390/ijms23073415 (PMC8998645; doi:10.3390/ijms23073415)
Supplement: Supplementary file 1 [file ijms-23-03415-s001.zip › Table S1.pdf]

	species	NCBI Assembly	Other websites
Ctenophora	*Monosiga brevicollis*	GCA_000002865.1V1.0	-
	Mnemiopsis leidyi	GCA_000226015.1MneLei_Aug2011	-
Porifera	*Amphimedon queenslandica*	GCA_000090795.1v1.0	-
Cnidaria	*Hydra vulgaris*	GCA_000004095.1Hydra_RP_1.0	-
	Acropora digitifera	GCA_000222465.2Adig_1.1	-
	Acropora millepora	GCA_013753865.1Amil_v2.1	-
	Discosoma sp	-	http://corallimorpharia.reefgenomics.org/download/
	Dendronephthya gigantea	GCA_004324835.1DenGig_1.0	-
	Pocillopora damicornis	GCA_003704095.1ASM370409v1	-
	Stylophora pistillata	GCA_002571385.1Stylophorapistillatav1	-
	Nematostella vectensis	GCA_000209225.1ASM20922v1	-
	Exaiptasia pallida	GCA_001417965.1Aiptasiagenome1.1	-
	Amplexidiscus fenestrafer	-	http://corallimorpharia.reefgenomics.org/download/
Xenacoelomorpha	*Hofstenia miamia*	-	https://metazoa.ensembl.org/Hofstenia_miamia/Info/Index
Lophotrochozoa	*Phoronis australis*	-	https://marinegenomics.oist.jp/pau_v2/viewer/download?project_id=51
	Notospermus geniculatus	-	https://marinegenomics.oist.jp/nge_v2/viewer/d-ownload?project_id=52

	Bugula neritina	GCA_010799875.2ASM1079987v2	-
	Lingula anatina	GCA_001039355.2LinAna2.0	-
	Acanthopleura granulata	-	https://alabama.app.box.com/s/1hsryfff61i01qrljyasrjnu8j7-qg2nj
	Octopus bimaculoides	GCA_001194135.1Octopus_bimaculoides_v2_0	-
	Octopus sinensis	GCA_006345805.1ASM634580v1	-
	Biomphalaria glabrata	GCA_000457365.1ASM45736v1	-
	Pomacea canaliculata	GCA_003073045.1ASM307304v1	-
	Elysia chlorotica	GCA_003991915.1ElyChl2.0	-
	Aplysia californica	GCA_000002075.2AplCal3.0	-
	Lottia gigantea	GCA_000327385.1Helro1	-
	Haliotis rufescens	GCA_003343065.1H.ruf_v1.0	-
	Haliotis laevigata	GCA_008038995.1Hlaev_1.0	-
	Scapharca broughtonii	-	https://www.protocols.io/view/the-pipeline-of-assembly-and-annotation-of-the-sca-z-7zf9p6
	Saccostrea glomerata	-	http://soft.bioinfo-minzhao.org/srog/#
	Crassostrea gigas	GCA_011032805.1ASM1103280v1	-
	Crassostrea virginica	GCA_002022765.4C_virginica-3.0	-
	Argopecten purpuratus	-	http://gigadb.or-g/dataset/view/id/100419
	Mizuhopecten yessoensis	GCA_002113885.2ASM211388v2	-

	Pinctada imbricata	GCA_002216045.1PinMar1.0	-
	Chlamys farreri	-	http://mgb.ouc.edu.cn/cfbase/html/
	Bathymodiolus platifrons	GCA_002080005.1Bpl_v1.0	https://datadryad.org/stash/dataset/doi:-10.5061/dryad.h9942
	Mytilus coruscus	GCA_017311375.1Mcoruscus_HiC	-
	Pinctada fucata	-	https://marinegenomics.oist.jp/pearl/viewer/download?project_id=3-6
	Capitella teleta	GCA_000328365.1Capca1	-
	Lamellibrachia luymesi	GCA_009193005.1LLUY_1.0	-
	Dimorphilus gyrociliatus	GCA_904063045.1Dgyrociliatus_assembly	-
	Eisenia foetida	GCA_003999395.1EiseniafetidaCLC_PE_MP	-
	Helobdella robusta	GCA_000326865.1Helobdellarobustav1.0	-
Ecdysozoa	*Daphnia pulex*	GCA_911175335.1PA424.2	-
	Drosophila melanogaster	GCA_000001215.4Release6plusISO1MT	-
	Tribolium castaneum	GCA_000002335.3Tcas5.2	-
	Penaeus vannamei	GCA_003789085.1ASM378908v1	-
Echinodermata	*Strongylocentrotus purpuratus*	GCA_000002235.4Spur_5.0	-
	Acanthaster planci	GCA_001949145.1OKI-Apl_1.0	-
Chordata	*Branchiostoma floridae*	GCA_000003815.2Bfl_VNyyK	-

	Ciona intestinalis	GCA_000224145.2KH	-
	Danio rerio	GCA_000002035.4GRCz11	-
	Xenopus tropicalis	GCA_000004195.4UCB_Xtro_10.0	-
	Gallus gallus	GCA_016699485.1bGalGal1.mat.broiler.GRCg7b	-
	Mus musculus	GCA_000001635.9GRCm39	-
	Myotis brandtii	GCA_000412655.1ASM41265v1	-
	Homo sapiens	GCA_000001405.28GRCh38.p13	-
