# Supplementary material for: Divergences of the RLR Gene Families across Lophotrochozoans: Domain Grafting, Exon–Intron Structure, Expression, and Positive Selection
Source: Int J Mol Sci. 2022 Mar 22;23(7):3415. doi: 10.3390/ijms23073415 (PMC8998645; doi:10.3390/ijms23073415)
Supplement: Supplementary file 1 [file ijms-23-03415-s001.zip › Table S2.pdf]

RLRs name	gene ID
ApuRLR1	XP_019854300.1
ApuRLR2	XP_003383379.1
AdiRLR1	XP_015762881.1
AdiRLR2	XP_015777225.1
AmiRLR1	XP_029213759.1
AmiRLR2	XP_029206383.1
DspRLR1	evm.model.scaffold_244.3
DspRLR2	evm.model.scaffold_294.23
DgiRLR1	XP_028414975.1
DgiRLR2	XP_028418574.1
PdmRLR1	XP_027039850.1
PdmRLR2	XP_027039857.1
PdmRLR3	XP_027052918.1
SpiRLR1	XP_022796607
SpiRLR2	XP_022806512.1
SpiRLR3	XP_022806513.1
NveRLR1	XP_032220115.1
NveRLR2	XP_001636291.2
EpaRLR1	XP_028516578.1
EpaRLR2	XP_028513750.1
EpaRLR3	XP_020896362.1
AfeRLR	evm.model.scaffold_105.34
PauRLR1	g1125
PauRLR2	g8212
NgeRLR1	g6120
NgeRLR2	g8783
NgeRLR3	g18494
NgeRLR4	g19819
NgeRLR6	g32542
NgeRLR7	g33830
BneRLR1	KAF6041430.1
BneRLR2	KAF6022551.1
LanRLR1	XP_013391115.1
LanRLR2	XP_013391116.1
LanRLR3	XP_013403651.1
LanRLR4	XP_013415811.1
LanRLR5	XP_023931160.1
LanRLR6	XP_013415812.1
LanRLR7	XP_013399073.1
LanRLR8	XP_013399861.1
AgrRLR1	model.g18212.t1_1
AgrRLR2	model.g18211.t1_1

AgrRLR3	model.g18206.t1_1
AgrRLR4	model.g21091.t1_1
AgrRLR5	model.g26263.t1_1
AgrRLR6	model.g26147.t1_1
AgrRLR7	model.g26286.t1_model.g26287.t1_1
ObiRLR	XP_014769581.1
OvuRLR	XP_029658486.1
BglRLR1	XP_013074823.1
BglRLR2	XP_013074821.1
BglRLR3	XP_013083291.1
BglRLR5	XP_013074848.1
PcaRLR1	XP_025094497.1
PcaRLR2	XP_025094498.1
PcaRLR3	XP_025095144.1
PcaRLR4	XP_025095736.1
PcaRLR5	XP_025096122.1
PcaRLR6	XP_025113860.1
EchRLR1	RUS70384.1
EchRLR2	RUS76323.1
EchRLR3	RUS76324.1
EchRLR4	RUS89371.1
AcaRLR1	XP_005090198.1
AcaRLR2	XP_005102833.1
AcaRLR3	XP_005104365.1
LgiRLR1	XP_009066516.1
LgiRLR2	XP_009058987.1
LgiRLR3	XP_009050249.1
HruRLR1	Halruf.G00034453
HruRLR2	Halruf.G00015009
HruRLR3	Halruf.G00031232
HruRLR4	Halruf.G00034449
HruRLR5	Halruf.G00034451
HruRLR6	Halruf.G00013690
HlaRLR1	HLAEV0016662
HlaRLR2	HLAEV0018699
HlaRLR3	HLAEV0043013
HlaRLR4	HLAEV0046281
HlaRLR5	HLAEV0035460
HlaRLR6	HLAEV0027544
SbrRLR1	EVM0023772.1
SbrRLR2	EVM0004967.1
SbrRLR3	EVM0010073.1
SbrRLR4	EVM0016435.1

SbrRLR5	EVM0011047.1
SglRLR1	Sgl012485-Mrna1
SglRLR2	Sgl027520-mRNA1
SglRLR3	Sgl027521-mRNA1
SglRLR4	Sgl001868-mRNA1
SglRLR5	Sgl010551-mRNA1
SglRLR6	Sgl003354-mRNA1
CgiRLR1	Cg08279
CgiRLR2	Cg08365
CgiRLR3	Cg08390
CgiRLR4	Cg08396
CgiRLR5	Cg20683
CgiRLR6	Cg20684
CgiRLR7	Cg22178
CgiRLR8	Cg22179
CgiRLR9	Cg23110
CgiRLR10	Cg23111
CgiRLR11	Cg23112
CgiRLR12	Cg23114
CgiRLR13	Cg24636
CviRLR1	XP_022333842.1
CviRLR2	XP_022288784.1
CviRLR3	XP_022339359.1
CviRLR4	XP_022304207.1
CviRLR5	XP_022339357.1
CviRLR6	XP_022293503.1
CviRLR7	XP_022339453.1
ApuRLR1	evm.model.scaffold_532.29
ApuRLR2	evm.model.scaffold_486.1
ApuRLR3	evm.model.scaffold_246.6.1
ApuRLR4	evm.model.scaffold_254.59
MyeRLR1	XP_021350240.1
MyeRLR2	XP_021351811.1
MyeRLR3	XP_021351834.1
MyeRLR4	XP_021369121.1
MyeRLR5	XP_021369135.1
PimRLR1	Pma_10010039
PimRLR2	Pma_10011360
PimRLR3	Pma_10014441
CfaRLR1	CF723635.1
CfaRLR2	CF42089.20
CfaRLR3	CF42089.21
BplRLR1	Bpl_scaf_42542-4.6

BplRLR2	Bpl_scaf_58803-1.42
BplRLR3	Bpl_scaf_42617-0.6
BplRLR4	Bpl_scaf_3477-1.8
BplRLR5	Bpl_scaf_58803-1.51
BplRLR6	Bpl_scaf_36154-0.2
BplRLR7	Bpl_scaf_4844-2.18
BplRLR8	Bpl_scaf_10347-0.27
BplRLR9	Bpl_scaf_58803-1.52
BplRLR10	Bpl_scaf_997-0.11
BplRLR11	Bpl_scaf_997-0.13
BplRLR12	Bpl_scaf_997-0.15
McoRLR1	CAC5370894.1
McoRLR2	CAC5377971.1
McoRLR3	CAC5388883.1
McoRLR4	CAC5388885.1
McoRLR5	CAC5394233.1
McoRLR6	CAC5394238.1
McoRLR7	CAC5397214.1
McoRLR8	CAC5397218.1
McoRLR9	CAC5401441.1
McoRLR10	CAC5408878.1
McoRLR11	CAC5409073.1
McoRLR12	CAC5409074.1
McoRLR13	CAC5409075.1
McoRLR14	CAC5414135.1
McoRLR15	CAC5418804.1
McoRLR16	CAC5422565.1
McoRLR17	CAC5422568.1
McoRLR18	CAC5422569.1
McoRLR19	CAC5422570.1
PfuRLR1	pfu_aug2.0_1600.1_01720.t1
PfuRLR2	pfu_aug2.0_841.1_04623.t1
PfuRLR3	pfu_aug2.0_841.1_04624.t1
PfuRLR4	pfu_aug2.0_303.1_10581.t1
CteRLR1	ELU14771.1
CteRLR2	ELU10263.1
CteRLR3	ELU11899.1
LluRLR1	LLU|FUN_004544-T1
LluRLR2	LLU|FUN_011577-T1
LluRLR3	LLU|FUN_014356-T1
DgyRLR	CAD5122599.1
EfoRLR1	evm.TU.Chr07.44
EfoRLR2	evm.TU.Chr09.644

EfoRLR3	evm.TU.Chr10.676
EfoRLR4	evm.TU.Chr10.677
EfoRLR5	evm.TU.Chr10.708
EfoRLR6	evm.TU.Chr11.411
EfoRLR7	evm.TU.Chr02.2952
HroRLR1	XP_009024874.1
HroRLR2	XP_009012916.1
SpuRLR1	XP_011665774.1
SpuRLR2	XP_030832117.1
SpuRLR4	XP_030841181.1
SpuRLR5	XP_030830677.1
SpuRLR7	XP_030830684.1
SpuRLR8	XP_030829778.1
SpuRLR9	XP_030830672.1
SpuRLR10	XP_030839787.1
SpuRLR11	XP_030840770.1
SpuRLR12	XP_783556.1
SpuRLR13	XP_030839793.1
SpuRLR14	XP_030840777.1
SpuRLR15	XP_030840874.1
SpuRLR16	XP_030832000.1
SpuRLR17	XP_030853694.1
SpuRLR18	XP_030853723.1
SpuRLR19	XP_030831983.1
AplRLR1	XP_022094377.1
AplRLR2	XP_022094379.1
AplRLR3	XP_022096807.1
AplRLR4	XP_022096855.1
AplRLR5	XP_022103897.1
AplRLR6	XP_022103922.1
BflRLR1	XP_035674578.1
BflRLR2	XP_035674522.1
BflRLR3	XP_035687787.1
BflRLR4	XP_035677687.1
BflRLR5	XP_019627228.1
BflRLR6	XP_019633355.1
BflRLR7	XP_035673073.1
CinRLR1	XP_018668656.1
CinRLR2	XP_002120168.3
DreRLR1	NP_001244086.1
DreRLR2	NP_001295492.1
DreRLR3	NP_001293024.1
XtrRLR1	XP_002935717.2

XtrRLR2	XP_002933320.3
XtrRLR3	XP_002939087.3
GgaRLR1	NP_001180567.1
GgaRLR2	NP_001305337.1
MmuRLR1	NP_001157949.1
MmuRLR2	NP_766277.3
MmuRLR3	NP_084426.2
MbrRLR1	XP_005875808.1
MbrRLR2	XP_014389648.1
MbrRLR3	XP_014393761.1
HsaRLR1	NP_071451.2
HsaRLR2	NP_055129.2
HsaRLR3	NP_077024.2
