# Supplementary material for: Divergences of the RLR Gene Families across Lophotrochozoans: Domain Grafting, Exon–Intron Structure, Expression, and Positive Selection
Source: Int J Mol Sci. 2022 Mar 22;23(7):3415. doi: 10.3390/ijms23073415 (PMC8998645; doi:10.3390/ijms23073415)
Supplement: Supplementary file 1 [file ijms-23-03415-s001.zip › Table S3.pdf]

	scaffold/chr	start	end	direction
NgeRLR1	scaffold134	236771	247817	-
NgeRLR2	scaffold220	100858	104643	+
NgeRLR3	scaffold717	22636	72343	-
NgeRLR4	scaffold805	204648	225701	-
NgeRLR6	scaffold2302	3268	24649	+
NgeRLR7	scaffold2548	41425	58662	+
Lan_RLR1	NW_019775380.1	1026135	1037218	+
Lan_RLR2	NW_019775380.1	1012307	1021957	+
Lan_RLR3	NW_019775457.1	183733	193909	+
Lan_RLR4	NW_019776013.1	142355	152774	-
Lan_RLR6	NW_019776013.1	123593	133070	-
Lan_RLR5	NW_019774456.1	3436	7522	-
Lan_RLR7	NW_019774203.1	580202	594420	+
Lan_RLR8	NW_019774569.1	230633	240561	-
AgrRLR1	Scaffold_100013	13146937	13163730	+
AgrRLR2	Scaffold_100013	13128319	13141943	+
AgrRLR3	Scaffold_100013	13076285	13094777	+
AgrRLR4	Scaffold_100017	928854	949170	+
AgrRLR5	Scaffold_100033	1816783	1833665	+
AgrRLR6	Scaffold_100033	144961	150130	+
AgrRLR7	Scaffold_100033	2119752	2140632	+
BglRLR1	NW_013246281.1	108539	119517	+
BglRLR2	NW_013246281.1	37608	52624	-
BglRLR3	NW_013246281.1	79010	101087	-
BglRLR4	NW_013391591.1	15374	46316	-
PcaRLR1	NC_037594.1	23240643	23254351	+
PcaRLR2	NC_037594.1	23256178	23266439	+
PcaRLR3	NC_037594.1	878058	893338	-
PcaRLR4	NC_037594.1	863532	876779	-
PcaRLR5	NC_037594.1	6215702	6255860	+
PcaRLR6	NC_037600.1	15229556	15240585	+
EchRLR1	scaffold001421	2964	>16663	+
EchRLR2	scaffold000671	40485	61274	+
EchRLR3	scaffold000671	66052	89736	-
EchRLR4	scaffold000058	662912	680995	+
HruRLR2	Scaffold_83	3236353	3254963	+
HruRLR3	Scaffold_289	246646	276145	+
HruRLR4	Scaffold_359	41403	59128	+
HruRLR5	Scaffold_359	77731	97120	+
HruRLR1	Scaffold_359	142164	169238	+
HruRLR6	Scaffold_77	2433187	2450509	+
Hla_RLR1	scaffold1944	130576	136385	-

Hla_RLR2	scaffold1891	86284	120730	+
Hla_RLR3	scaffold17787	15010	22709	-
Hla_RLR4	scaffold17381	11580	14366	+
Hla_RLR5	scaffold9256	17963	39210	-
Hla_RLR6	scaffold3353	57977	63842	+
Sbr_RLR1	Lachesis_group12	16184551	16211436	-
Sbr_RLR2	Lachesis_group12	40752732	40776429	-
Sbr_RLR3	Lachesis_group15	33393981	33411759	-
Sbr_RLR4	Lachesis_group16	26650043	26670076	-
Sbr_RLR5	Lachesis_group8	19301388	19328272	-
SglRLR1	SGL_77	808918	857653	+
SglRLR2	SGL_1805	32075	36386	-
SglRLR3	SGL_1805	46007	47747	-
SglRLR4	SGL_3	389359	396624	+
SglRLR5	SGL_48	1707872	1721522	+
SglRLR6	SGL_25	2098886	2121144	+
CgiRLR1	CHR03	26752659	26766810	-
CgiRLR2	CHR03	25858662	25863427	+
CgiRLR3	CHR03	26690111	26717721	-
CgiRLR4	CHR03	27471599	27482127	+
CgiRLR5	CHR07	13267581	13274169	-
CgiRLR6	CHR07	13275261	13325384	-
CgiRLR7	CHR07	41418057	41430676	-
CgiRLR8	CHR07	41443515	41461911	-
CgiRLR9	CHR08	1062613	1054628	+
CgiRLR10	CHR08	1049880	1032826	+
CgiRLR11	CHR08	1011828	1010483	+
CgiRLR12	CHR08	1000082	992574	+
CgiRLR13	CHR08	28581780	28595182	-
CviRLR1	NC_035780.1	7520664	7533035	-
CviRLR2	NC_035785.1	32081636	32100147	-
CviRLR3	NC_035784.1	67021428	67028280	-
CviRLR4	NC_035780.1	7504984	7518262	-
CviRLR5	NC_035784.1	67006508	67020882	-
CviRLR6	NC_035786.1	1030784	1040720	+
CviRLR7	NC_035780.1	55290877	55309979	+
ApuRLR1	scaffold_532	879983	896220	-
ApuRLR2	scaffold_486	13021	29216	+
ApuRLR3	scaffold_246	165108	185426	+
ApuRLR4	scaffold_254	986654	1000805	+
MyeRLR1	NW_018403348.1	17229	21410	+
MyeRLR2	NW_018403549.1	277903	299058	-
MyeRLR3	NW_018403549.1	241210	274388	-

MyeRLR4	NW_018408534.1	102736	144402	-
MyeRLR5	NW_018408534.1	150703	215618	+
BplRLR1	Bpl_scaf_42542	341855	366901	-
BplRLR2	Bpl_scaf_58803	175502	208748	-
BplRLR3	Bpl_scaf_42617	25153	36676	+
BplRLR4	Bpl_scaf_3477	111353	136658	+
BplRLR5	Bpl_scaf_58803	136738	152330	+
BplRLR6	Bpl_scaf_36154	2738	6174	-
BplRLR7	Bpl_scaf_4844	237084	263808	-
BplRLR8	Bpl_scaf_10347	50734	58988	+
BplRLR9	Bpl_scaf_58803	165812	172160	-
BplRLR10	Bpl_scaf_997	74877	82916	-
BplRLR11	Bpl_scaf_997	953	12641	-
BplRLR12	Bpl_scaf_997	33887	66067	-
McoRLR1	CACVKT020001740.1	547689	570392	+
McoRLR2	CACVKT020002442.1	592928	630507	+
McoRLR3	CACVKT020004272.1	51499	94552	+
McoRLR4	CACVKT020004272.1	118320	122508	-
McoRLR5	CACVKT020005265.1	1788380	1800074	+
McoRLR6	CACVKT020005265.1	1918449	1937875	+
McoRLR7	CACVKT020005668.1	104988	139777	-
McoRLR8	CACVKT020005668.1	276017	297407	-
McoRLR9	CACVKT020006413.1	21147	53458	-
McoRLR10	CACVKT020007611.1	5605	31363	+
McoRLR11	CACVKT020007631.1	78109	106480	-
McoRLR12	CACVKT020007631.1	116290	134539	-
McoRLR13	CACVKT020007631.1	161816	177451	-
McoRLR14	CACVKT020008322.1	306532	323234	-
McoRLR15	CACVKT020008950.1	628386	653638	+
McoRLR16	CACVKT020009642.1	422340	453634	-
McoRLR17	CACVKT020009642.1	472665	501192	+
McoRLR18	CACVKT020009642.1	532099	575031	+
McoRLR19	CACVKT020009642.1	591503	621434	+
PfuRLR1	scaffold1600.1	60475	83845	-
PfuRLR2	scaffold841.1	264309	279424	+
PfuRLR3	scaffold841.1	285579	322259	+
PfuRLR4	scaffold303.1	32566	52303	+
EfoRLR1	GWHACBE00000007	1041111	1049606	-
EfoRLR2	GWHACBE00000009	26265846	26304445	-
EfoRLR3	GWHACBE00000010	25834444	25869441	-
EfoRLR4	GWHACBE00000010	25893524	25912328	-
EfoRLR5	GWHACBE00000010	27347210	27364339	-
EfoRLR6	GWHACBE00000011	11985053	12017817	-

EfoRLR7	GWHACBE00000002	106328332	106339368	+
