# Supplementary material for: Divergences of the RLR Gene Families across Lophotrochozoans: Domain Grafting, Exon–Intron Structure, Expression, and Positive Selection
Source: Int J Mol Sci. 2022 Mar 22;23(7):3415. doi: 10.3390/ijms23073415 (PMC8998645; doi:10.3390/ijms23073415)
Supplement: Supplementary file 1 [file ijms-23-03415-s001.zip › Table S4.pdf]

1. *Phoronis australis*

	LP	TK	AM
RLR1-V2	9.04533	3.99136	2.81048
RLR2-L2	7.50413	7.62317	10.1614

2. *Notospermus geniculatus*

	PB	AE	AP1	AP2	MB	PE
R6-C	9.81139	6.0322	7.08243	7.03829	3.56598	6.07398
R7-C	18.0409	16.3931	15.37	16.2688	12.5438	17.0937
R3-V2	1.08933	4.23435	3.55082	2.55776	2.19924	2.59355
R4-V1	7.66755	6.321	4.64242	5.73094	3.8712	6.84698
R1-L5	3.65839	4.64891	7.66877	3.75824	5.54975	5.23155
R2-L6	26.6076	39.3768	36.075	18.0152	17.2067	52.407

3. *Lingula anatine*

	L	WG	DC	DM	VM
Lan_RLR1	10.8757	10.6239	39.0412	6.73605	6.24815
Lan_RLR3	4.27099	4.02718	7.00845	2.6681	1.99204
Lan_RLR2	10.5772	9.26149	11.5139	5.15105	4.4413
Lan_RLR5	1.111	2.31656	5.39354	1.08909	0.906858
Lan_RLR7	8.90447	0.864745	0.080344	2.66162	2.25311
Lan_RLR4	2.00301	1.3098	0.885741	0.754496	0.487079
Lan_RLR6	1.44662	3.02376	5.24837	1.20844	0.929965
Lan_RLR8	0.599738	1.18154	2.22934	0.478682	0.390179

4. *Octopus bimaculoides*

	ST	E	OO	SM	BO
ObiRLR-V2	15.3904	18.5358	125.986	29.2199	17.5995

5. *Haliotis rufescens*

	liver	Man	heart	Fgo	gan	gill
Hru_RLR1	2.79817	4.145545	11.54822	1.304403	1.694371	22.05185
Hru_RLR2	1.84014	0.225214	1.014892	0.166692	0.140119	1.245273
Hru_RLR4	2.43847	0.388523	1.035464	0.468846	0	1.160215
Hru_RLR3	2.44257	1.8718	4.38007	0.832851	0.240173	5.532295
Hru_RLR6	7.606065	2.11973	5.791305	0.776802	1.078369	6.612095
Hru_RLR5	3.450625	0.261792	1.972844	0.167591	0.313006	1.447935

6. *Crassostrea gigas*

	Fgo	Man	Lpa	Dgl	Gil	Amu	Hem	Mgo
R12-C	0.585713	2.01651	2.22463	0	10.0576	8.93197	18.4033	4.50188
R1-V2	0.560081	0.701199	3.24707	0	0	0	0	0.986553
R2-V2	0.314552	1.31186	17.9888	11.2609	0	0	0	0.754886
R4-V2	1.4324	1.9926	4.63148	3.78148	3.03589	0	0	4.12031

R5-V2	6.56991	8.99491	13.0176	11.722	4.70532	0	0	48.2519
R3-V1	0.8947	2.13824	5.65756	4.11013	0	0	0	5.94016
R7-V1	19.1142	44.6061	39.7868	27.5755	38.7414	29.4912	42.5332	16.5349
R8-V1	7.36036	17.9063	14.5647	19.3169	8.86193	11.8051	17.0263	9.43042
R9-V1	11.9927	20.6565	13.8345	16.1312	12.9506	17.2518	10.6636	22.6915
R10-V1	0	0	0.07445	0	0	0	0	5.50129
R13-V1	14.969	25.9724	27.5581	7.33566	7.8525	7.84527	6.46579	14.8191
R6-X2	9.26362	15.7115	12.3029	14.5349	5.83465	7.77232	4.80428	11.9562
R11-L6	0	0	0	0	0	19.694	0	0

7. *Mytilus coruscus*

	Mou	Mal	Hae	Gut	Gill	Foo	Fem	Dig	Add
R1-V2	7.31762	0.84356	4.37648	1.79655	6.78631	2.69678	2.73885	4.72127	1.0986
R2-V2	2.61141	0.321471	22.6316	4.19373	1.39515	0.186578	1.25125	5.98751	1.02067
R11-V2	0.888494	1.68092	1.47917	2.13602	0.994409	1.06282	0.371402	1.84721	0.923981
R16-V1	0	0.059415	0.091882	0	0.090955	0.722616	0.115466	0	0
R17-V1	4.78841	4.12659	2.14618	2.70179	5.90234	1.17581	6.90289	2.38932	4.78832
R18-V1	3.5929	9.31875	5.82299	9.93981	4.44178	4.07133	4.60835	3.11006	2.2136
R19-V1	20.4432	7.35841	0.843567	8.48995	21.0712	4.2633	9.8671	4.433	4.23464
R3-L6	0	0.478	2.25302	0	0.916665	2.093	0	3.1364	1.67318
R4-L6	2.17517	1.83434	2.87302	2.1158	1.87533	0.356413	3.77899	0.381431	2.13426
R5-L4	2.93538	0	3.80578	1.61704	1.38733	0.371057	0.622113	0	0.369057
R6-L4	0	0	0	0	0	0	0	0	0

R7-L4	0.428817	0.538513	0	0	0.515705	0	0.393326	0.753269	0.233663
R8-L4	0	0.406501	0.314922	0.064938	0	0	1.02765	0	0
R9-L4	0.086911	0	0	0	0.062918	0.142925	0	0	0
R12-L4	7.44394	1.21611	3.02977	12.35	3.72459	1.51109	0.422375	3.07092	1.65211
R13-L4	9.72594	6.26681	21.2486	5.52564	14.7057	8.35119	2.76973	3.78485	10.5494
R14-L4	0.347645	0.493066	0	0.525111	0.755016	1.42925	0.239717	1.37583	0
R15-L4	0	0	0	0	0	0	0	0	0
R10-X4	9.55227	3.64989	3.27884	28.6046	4.80485	3.97313	13.0992	26.9889	1.97638

8. *Mizuhopecten yessoensis*

	Kid	SM	Gill	Man	Gon	Hem
R2-A1	17.5728	31.8591	27.3893	15.89453	4.0534	53.4057
R3-A1	14.29956	19.99418	15.94991	13.83281	3.733653	55.0217
R4-V1	2.306807	2.672923	1.965613	1.107919	1.741431	3.821405
R5-V1	7.89571	10.24286	6.48064	5.47577	6.570887	7.56127
R1-L6	5.017643	7.939403	5.205947	1.454188	1.027113	6.886925

9. *Chlamys farreri*

	Gill	Kid	SM	Man	Fgo	Mgo
R2-C	2.176283	2.136445	2.58631	2.191623	1.161762	1.696363
R3-V1	3.581133	3.083237	5.288096667	2.913087	1.60417	0.758247
R1-L6	1.12095	0.369498	0.394543333	0.537071	0.230801	0

10. *Eisenia foetida*

	NC	CG	C	G	P	CF
R3-V2	7.11634	13.5192	3.33603	7.80827	25.0207	2.23838
R4-V2	0.239653	0	0	0	4.4352	0
R2-V1	4.05991	6.77071	6.62332	5.52377	9.19854	2.38559
R6-V1	0.917901	1.1085	1.48441	1.58187	2.52953	0.241014
R1-L2	10.4654	6.39507	9.91135	7.31209	7.05815	9.48339
R5-L2	2.8334	2.3304	3.12432	3.48795	6.20729	1.69273
R7-L2	1.70627	1.93092	1.91625	1.96913	2.01994	1.12055
