# Supplementary figures and images for: Divergences of the RLR Gene Families across Lophotrochozoans: Domain Grafting, Exon–Intron Structure, Expression, and Positive Selection
Source: Int J Mol Sci. 2022 Mar 22;23(7):3415. doi: 10.3390/ijms23073415 (PMC8998645; doi:10.3390/ijms23073415)

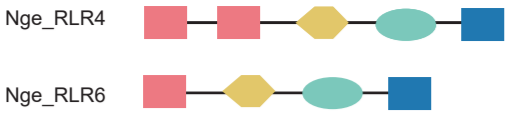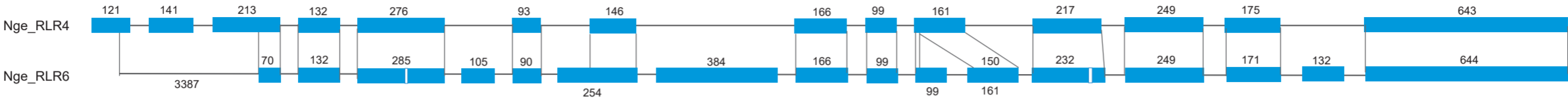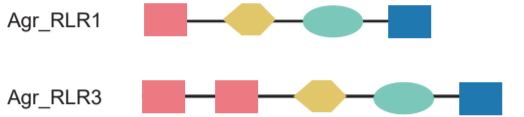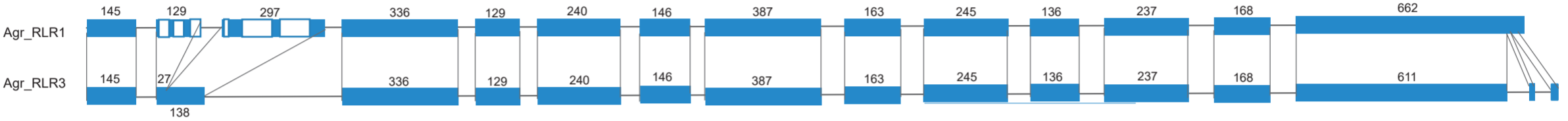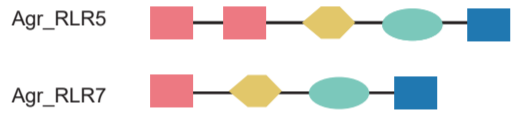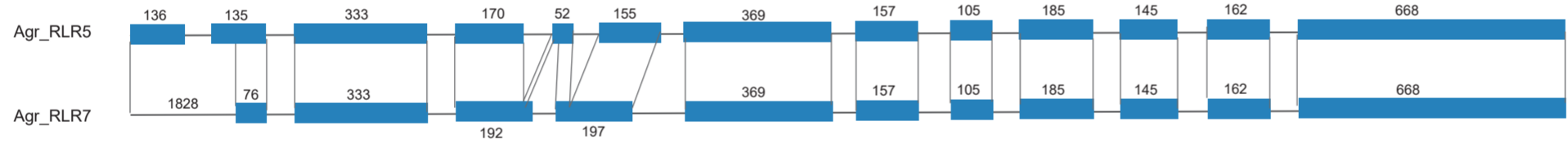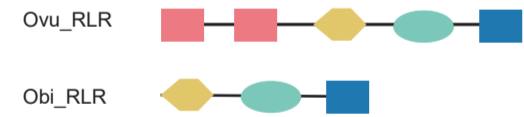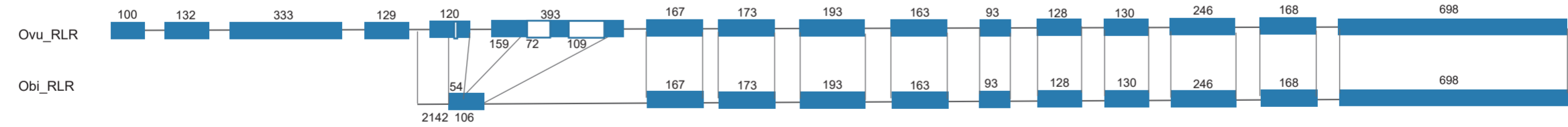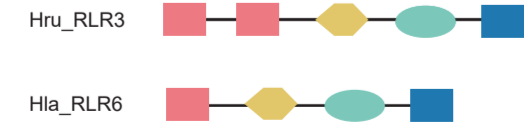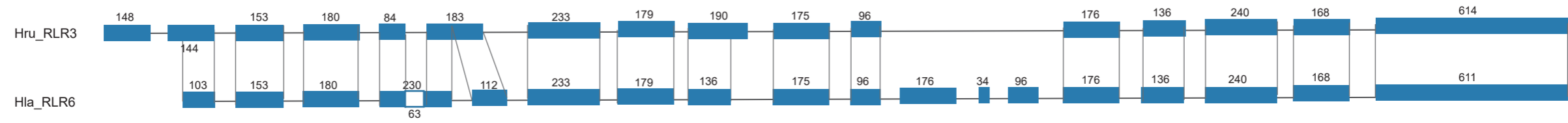

Supplement: Supplementary file 1 [file ijms-23-03415-s001.zip › Figure S1.pdf]
